# Nigrostriatal Dopaminergic Dysfunction and Altered Functional Connectivity in REM Sleep Behavior Disorder With Mild Motor Impairment

**DOI:** 10.3389/fneur.2019.00802

**Published:** 2019-07-26

**Authors:** Gohei Yamada, Yoshino Ueki, Naoya Oishi, Takuya Oguri, Ayako Fukui, Meiho Nakayama, Yuko Sano, Akihiko Kandori, Hirohito Kan, Nobuyuki Arai, Keita Sakurai, Ikuo Wada, Noriyuki Matsukawa

**Affiliations:** ^1^Department of Neurology, Nagoya City University Graduate School of Medical Science, Aichi, Japan; ^2^Department of Rehabilitation Medicine, Nagoya City University Graduate School of Medical Science, Aichi, Japan; ^3^Medical Innovation Centre, Kyoto University Graduate School of Medicine, Kyoto, Japan; ^4^Department of Neurology, Tosei General Hospital, Aichi, Japan; ^5^Department of Otolaryngology and Good Sleep Centre, Nagoya City University Graduate School of Medicine, Aichi, Japan; ^6^Research & Development Group, Centre for Technology Innovation - Healthcare, Hitachi Ltd, Saitama, Japan; ^7^Department of Radiology, Nagoya City University Hospital, Aichi, Japan; ^8^Department of Radiology, Teikyo University, Tokyo, Japan

**Keywords:** REM sleep behavior disorder, finger tapping, SPECT, resting state functional MRI, dopaminergic dysfunction, functional connectivity

## Abstract

Rapid eye movement sleep behavior disorder is parasomnia characterized by symptoms of dream enactment and loss of muscle atonia during rapid eye movement sleep. Mild motor impairment is present in some patients with rapid eye movement sleep behavior disorder and presumed to be a risk factor for conversion to synucleinopathies. The purpose of this study is to identify patients with mild motor impairment by evaluating finger tapping and to investigate its pathophysiology. Twenty-three patients with rapid eye movement sleep behavior disorder and 20 healthy control subjects were recruited in the present study. We accurately evaluated finger tapping including amplitude, peak open, and close speed with a magnetic sensing device and identified patients with mild motor impairment. Moreover, we performed ^123^I-2β-carbomethoxy-3β-(4-iodophenyl) nortropane SPECT and resting state functional MRI. ^123^I-2β-carbomethoxy-3β-(4-iodophenyl) nortropane uptake for each bilateral caudate, anterior putamen, and posterior putamen was calculated and the resting state functional connectivity of sensorimotor network was analyzed. Using finger tapping parameters, we identified eight patients with mild motor impairment. In patients with mild motor impairment, all finger tapping parameters were significantly impaired when compared to patients with normal motor function, while they exhibited no significant differences in Unified Parkinson's Disease Rating Scale part III score. ^123^I-2β-carbomethoxy-3β-(4-iodophenyl) nortropane uptake in the right posterior putamen, bilateral anterior putamen, and caudate was significantly lower when compared to healthy controls or patients with rapid eye movement sleep behavior disorder with normal motor function. These patients also exhibited decreased cortico-striatal functional connectivity and increased cortico-cerebellar functional connectivity when compared to healthy controls or patients with normal motor function. Our results show that mild motor impairment in rapid eye movement sleep behavior disorder evaluated by finger tapping task presented mild nigrostriatal dopaminergic dysfunction as well as alterations in resting state sensorimotor network. Although longitudinal follow up is necessary, such patients may have higher risk of short-term conversion to synucleinopathies.

## Introduction

REM sleep behavior disorder (RBD) is parasomnia characterized by symptoms of dream enactment and loss of muscle atonia during REM sleep. Previous studies revealed that RBD precedes the clinical onset of neurodegenerative diseases, particularly synucleinopathies (1–6). Although mild motor impairment in patients with RBD is one predictor of the conversion from RBD to Parkinson's disease or dementia with Lewy bodies (5, 6), its pathophysiology remains unclear.

In the present study, we attempted to identify patients with RBD with mild motor impairment using quantitative evaluation of finger tapping, and to clarify its pathophysiology using dopamine transporter imaging and resting state functional MRI (rsfMRI). Finger tapping is a useful maneuver to assess parkinsonism (7–9), and its parameters such as amplitude and speed can be quantitatively evaluated using a magnetic sensing device (10). In the previous study of Parkinson's disease, the patient group (patients with mild to moderate motor impairment, UPDRS-III score 19.2 ± 7.8) presented lower amplitude, lower opening speed, and lower closing speed in finger tapping compared to the control group (7). By using finger tapping speed and amplitude, motor dysfunction whose pathophysiological mechanisms are similar to Parkinson's disease can be detectable in RBD patients.

To investigate brain function in patients with RBD with mild motor impairment, we performed two neuroimaging methods. One is dopamine transporter imaging and another is resting state functional MRI. Nigrostriatal dopaminergic neurons are progressively degenerated in synucleinopathies such as Parkinson's disease and dementia with Lewy bodies, which is reflected by the reduction of ^123^I-2β-carbomethoxy-3β-(4-iodophenyl) nortropane (^123^I-FP-CIT) uptake in the striatum (11–13). The loss of nigrostriatal dopaminergic neuron is associated with motor dysfunction in patients with Parkinson's disease (14, 15). We speculated that mild motor impairment was induced by subclinical nigrostriatal dopaminergic dysfunction. The reduction of ^123^I-FP-CIT uptake can also identify patient groups at high risk of short-term conversion from RBD to synucleinopathies (16, 17). Resting state functional MRI (rsfMRI) is a method to identify functionally connected brain regions using blood oxygen level dependent signal at rest (18). In Parkinson's disease, rsfMRI revealed decreased functional connectivity between the posterior putamen and inferior parietal cortex (19), increased functional connectivity between the motor cortex and cerebellum (20), and reduced functional connectivity between striatum and thalamus (21). In RBD, previous research suggested the reduction of functional connectivity in basal ganglia network, which begins earlier than nigrostriatal dopaminergic dysfunction, could serve as a possible biomarker of the prodromal stage of Parkinson's disease (22, 23). The aim of this study is to identify patients with RBD with mild motor impairment using finger tapping parameters and to clarify its pathophysiology using ^123^I-FP-CIT SPECT and rsfMRI.

## Materials and Methods

### Participants

Twenty-three RBD patients (12 male and 11 female, age 71.3 ± 4.2 years) and 20 age- and sex-matched healthy control (HC) subjects (11 male and 9 female, age 70.7 ± 3.6 years) were recruited in this study at Nagoya City University Hospital between January 2015 and March 2018. All subjects were right-handed. Patients were diagnosed with RBD based on the Internal Classification of Sleep Disorders 3rd edition, which includes a history of dream enactment with complex motor movements and the presence of increased tonic or phasic electromyography activity during REM sleep (24). Every patients underwent video-polysomnography. The duration of RBD was calculated as the time from when patients first noticed symptoms. Exclusion criteria included RBD secondary to brain structural abnormalities, medication use, Parkinson's disease, dementia with Lewy bodies or multiple systemic atrophy, disabilities that restricted finger movements, standard contraindications to MRI scanning, dementia, and other neurologic or psychiatric diseases. All participants completed Edinburgh Handedness Inventory, Unified Parkinson Disease Rating Scale (UPDRS) part III, Mini-Mental State Examination (MMSE), Japanese version of RBD Screening Questionnaire (RBDSQ-J) and “Open Essence” (a card-type odor identification test) (25), Epworth Sleepiness Scale, Hospital Anxiety and Depression Scale (HADS), HADS-anxiety, HADS-depression, and Pittsburgh Sleep Quality Inventory. The study protocol was approved by the Ethics Committee of the Nagoya City University. All subjects provided written informed consent before starting the study. This study was performed according to the ethical guidelines established by Declaration of Helsinki.

### Finger Tapping

**Figure 1:**
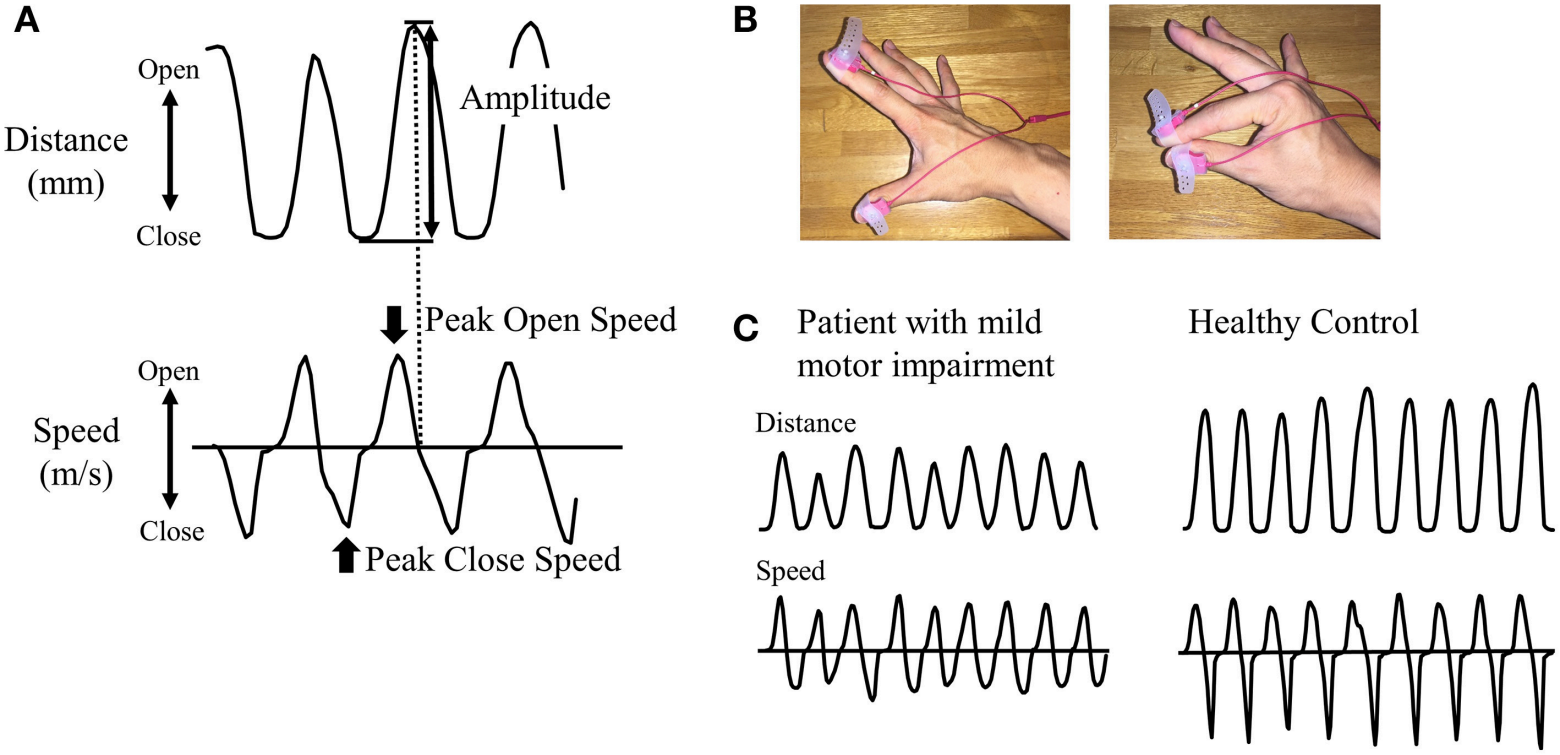
Recording of finger tapping. **(A)** As parameters, amplitude, peak open speed, and peak close speed were recorded for each cycle of finger tapping. **(B)** Magnetic sensors were attached to the index finger and thumb. The left panel shows the opening position of fingers and the right panel shows the closing position of fingers. **(C)** Representative examples of distance and speed are shown for patients in RBD-MMI group and healthy control subjects.

A magnetic sensing finger tapping device (UB-1; Finger Tapping Movement Analyzer; Hitachi Computer Peripherals Co., Ltd., Kanagawa, Japan) was used to record finger movement (26). The sensors of an electromagnetic tracking device were attached to the thumb and index finger of each subject (Figure 1). Subjects were then instructed to tap their index finger and thumb by each hand as widely and rapidly as possible for 15 s; third, fourth, and fifth digits remained in a semi-extended position during finger tapping. The order of the right and left finger tapping tasks was pseudorandomized.

### Kinetic Parameters and the Classification of Patients With RBD

We measured the amplitude (mm) and peak open speed and close speeds (m/s) for each cycle of finger tapping (Figure 1). Mean values of each parameter during 15-s tap cycles were calculated. Based on finger tapping parameters, patients with RBD were classified into two groups: RBD with mild motor impairment (RBD-MMI) and RBD with normal motor function (RBD-N). Since there was no previous study to define mild motor impairment using finger tapping parameters, we originally defined the criteria for mild motor impairment. In order to be classified into the RBD-MMI group, at least one in six parameters, including bilateral amplitude and peak open and peak close speed, had to be less than two standard deviation (SD) of the mean value of the HC group. Patients with RBD who did not fulfill these criteria were classified into the RBD-N group. Considering the influence of dominant-hand or not, we separately defined the cut-off values in the right and left side. Moreover, to investigate the pattern of the reduction in the finger tapping parameters (progressive decrement or consistent lower value), we calculated the slope of the fitted linear regression line across the finger tapping parameters against the tap cycle. We defined the progressive decrement as the slope value, calculated as less than 2 SD of the mean value of the HC group.

### Imaging Data Acquisition

#### ^123^I-FP-CIT SPECT

Twenty-two patients with RBD and 12 HC subjects were included in the analysis for ^123^I-FP-CIT SPECT. For evaluating dopaminergic nigrostriatal neurons (27–29), ^123^I-FP-CIT SPECT was performed to measure the concentration of dopamine reuptake transporters within the striatum and cerebellum for 22 out of 23 patients with RBD and 12 out of 20 HC subjects. ^123^I-FP-CIT SPECT was performed using a dual-head scintillation camera (E-CAM, Toshiba, Tokyo, Japan) with a high-resolution fan beam collimator, 3 h after a bolus injection of 167 MBq of ^123^I-FP-CIT (provided as DaTSCAN; Mediphysics, Tokyo, Japan). Imaging time was 30 min and performed following the ioflupane guideline working group recommendation proposed by the Japanese Society of Nuclear Medicine (Available from http://www.jsnm.org/archives/1151/) (acquisition parameters: 90 views with a 128 × 128 matrix and 1.45 zoom, 2.3 s per view in the continuous mode; 20% energy window centered on 159 keV photopeak of ^123^I; 2 million total counts/per acquisition). Image reconstructions were created using the ordered subsets expectation maximization algorithm. Reconstructed images were smoothed using a 3D Gaussian filter (full-width at half maximum, 0.70 cm).

#### Brain MRI

Data acquisition was performed at Nagoya City University hospital using a 3T MRI scanner (MAGNETOM Skyra, Siemens, Erlangen, Germany) with a 32-channel head matrix coil. T1-weighted images were taken using a 3D magnetization prepared-rapid acquisition gradient echo (MPRAGE) sequence (repetition time/echo time/inversion time = 2,300/2.96/900 ms, flip angle = 9 degrees, matrix size = 256 × 256, field of view = 256 mm, slice thickness = 1.0 mm) for registration purposes.

Whole-brain rsfMRI scans were acquired with single-shot gradient echo planar imaging (GE-EPI). (repetition time/echo time = 2,500/30 ms, flip angle = 80 degrees, resolution = 3 × 3 × 3 mm). Thirty-nine axial slices were acquired per volume, covering both hemispheres and the cerebellum. One-hundred and eighty repetitions were acquired in about 7.5 min. During functional MR imaging, participants were instructed to remain still and stare at a cross sign on the monitor screen. After that, participants were asked whether they could comply with the instruction. Distortions were corrected using a (B_0_) field map calculated from the phase change between images with different echo times (TEs).

## Data Analysis

### Clinical and Demographic Data

Comparisons of continuous variables were carried out by univariate ANOVA. Tukey's honesty significance difference *post-hoc* analysis was used to determine differences between RBD-MMI, RBD-N, and HC group. A two-sample *t*-test was used to compare polysomnographic measures and disease durations between RBD-MMI and RBD-N group. A chi-square test was used for discreet variables. Statistical analyses were performed using commercially available software (SPSS Ver.18.0) and a two-tailed *P*-value of < 0.05 was considered significant.

### ^123^I-FP-CIT SPECT

Image processing was performed using SPM8 (Wellcome Trust Centre for Neuroimaging, London, UK) under MATLAB 2014a for Windows (MathWorks, Natick, MA). 3D T1-weighted MRI was coregistered to the reconstructed SPECT image of the individual subjects using a rigid transformation. The MRI was spatially normalized to the standard Montreal Neurological Institute (MNI) space with non-linear warping in order to obtain an inverse deformation field using VBM8 Toolbox (http://dbm.neuro.uni-jena.de/vbm). The caudate nucleus, putamen, and cerebellum were selected as volumes of interests (VOIs). These VOIs were created based on the Automated Anatomical Labeling (AAL) template (30), and the putamen was separated into anterior (y < 0) and posterior (y ≥ 0) aspects based on a previous report (31). All VOIs in the standard MNI space were inversely transformed to individual spaces by SPM8 using the inverse deformation field. Since these individual VOIs were automatically defined, the operator-induced bias that is often introduced by defining VOIs manually was avoided (32). ^123^I-FP-CIT uptake was defined as the specific binding ratio [(striatal counts-cerebellar counts)/cerebellar counts] (33), and was calculated for the bilateral caudate, as well as anterior and posterior putamen. The asymmetry of binding between bilateral sides was expressed as an asymmetry index: which was defined as |R-L|/(R+L).

Differences in the tracer uptake and asymmetry index of each region between RBD-MMI, RBD-N, and HC group were evaluated using a univariate ANOVA. A *post-hoc* Tukey test was used to determine differences between groups. A two-tailed *P*-value < 0.05 was considered significant.

### RsfMRI

EPI data were undistorted with FMRIB's Utility for Geometrically Unwarping EPIs in the FSL package. Functional images were preprocessed and analyzed with the CONN-fMRI toolbox (17f) (34) and SPM12 (Wellcome Trust Centre for Neuroimaging, London, UK). Pre-processing steps included slice-timing correction, realignment, Artifact Detection Tools-based identification of outlier scans for scrubbing (35), segmentation and normalization to the MNI space, and spatial smoothing with an 8-mm Gaussian kernel of full-width at half maximum and band-pass filtering (0.008 < f < 0.09 Hz) (36). The removed temporal confounding factors included realignment parameter noises and BOLD signals from white matter and cerebrospinal fluid.

Individual regions of interest (ROIs) were defined to study resting state sensorimotor networks. Seed ROIs were defined as follows: primary motor cortex (M1), premotor cortex (PM), supplementary motor area (SMA), primary sensory cortex (S1), superior parietal lobule (SPL), inferior parietal lobule (IPL), caudate nucleus, anterior putamen, posterior putamen, cerebellar lobules II, III, IV–V, VI, VII, and VIII. Seed regions, except for M1, S1, and PM, were based on the AAL atlas (30). Seed regions including M1 and S1 were created using the Brodmann atlas. The ROI of the PM was created using the AAL and Brodmann atlases. Correlation coefficients (bivariate) were calculated between the BOLD time series signal of each ROI and those of every other ROI. Fisher-z-transformation was applied to convert the resulting correlation coefficients. Differences in functional connectivity among the three groups were determined using age and sex as covariates. The threshold for significance was a FDR-adjusted *P*-value of < 0.05 (two-tailed). Absolute head motion during functional MRI acquisitioning was assessed with the volume-based frame-wise displacement (35). Differences in absolute head motion between RBD-MMI, RBD-N, and HC groups were evaluated using a univariate ANOVA. A *post-hoc* Tukey test was used to determine differences between groups. A two-tailed *P*-value of < 0.05 was considered significant.

### Correlation Analysis

#### Correlation Between ^123^I-FP-CIT Uptake and Finger Tapping or UPDRS-III Score

We used Pearson's correlation coefficients between finger tapping parameters or UPDRS-III score (total score and finger tapping score) and ^123^I-FP-CIT uptake in the contralateral striatum in all subjects and used a threshold (two-tailed *P* < 0.05) to determine significance.

#### Correlation Between Resting State Functional Connectivity and Finger Tapping or UPDRS-III Score

We used a partial correlation, controlling for age and sex, between finger tapping parameters or UPDRS-III score (total score and finger tapping score) and functional connectivity coefficients showing a significant group difference in all subjects and used a threshold (two-tailed *P* < 0.05) to determine significance.

## Results

### Finger Tapping

**Figure 2:**
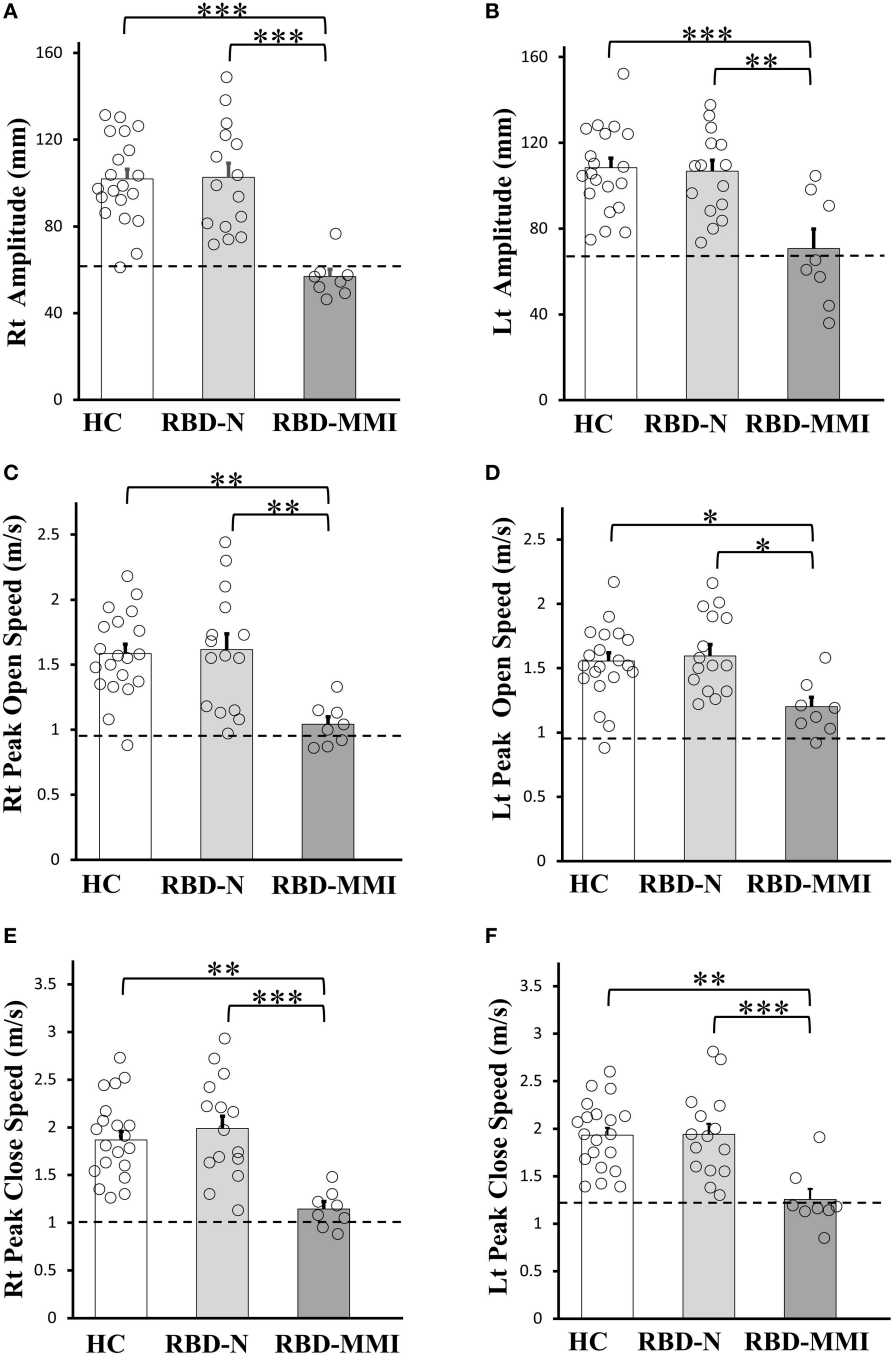
Comparisons of the finger tapping task among RBD-MMI, RBD-N, and HC group. Right and left amplitude **(A,B)**, peak open speed **(C,D)**, and peak close speed **(E,F)** were significantly lower in patients of the RBD-MMI group when compared to those in the RBD-N and HC group. The bar chart illustrates the mean amplitude (mm), peak open speed, and peak close speed (m/s). Error bars represent the standard error of the mean. Dashed lines represent−2 SDs. *P*-values were corrected using Tukey's multiple comparison test. ^*^P < 0.05, ^**^P < 0.01, ^***^P < 0.001.

Results of finger tapping are shown in Figure 2. According to our classification criteria, eight (36%) patients belonged to the RBD-MMI group and 14 patients belonged to the RBD-N group. Seven patients exhibited right amplitude that was <2 SD of the mean value of the right amplitude of the HC group. Five patients exhibited left amplitude that was less than 2 SD of the mean value of the left amplitude of the HC group. Three patients exhibited right open speed that was less than 2 SD of the mean value of the right open speed of the HC group. One patient exhibited left open speed that was <2 SD of the mean value of the left open speed of the HC group. Two patients exhibited right close speed that was <2 SD of the mean value the right close speed of the HC group. Six patients exhibited left close speed that was <2 SD of the mean value of the left close speed of the HC group. A univariate ANOVA with a *post-hoc* Tukey test showed all finger tapping parameters were significantly reduced in the RBD-MMI group when compared to the RBD-N and HC groups. There was no significant difference between the RBD-N and HC groups.

Based on the mean slope of finger tapping parameters in the HC control group, we investigated the pattern of lower amplitude, open speed, and close speed in eight patients with MMI. The slope of amplitude in the HC group was −0.17 ± 0.28 in the right side and −0.31 ± 0.32 in the left side. The slope of open speed in the HC group was −0.009 ± 0.007 in the right side and −0.013 ± 0.006 in the left side. The slope of close speed in the HC group was −0.012 ± 0.008 in the right side and −0.016 ± 0.009 in the left side. No patient in the RBD-MMI group exhibited a slope value that was less than 2 SD of the mean slope value measured in the HC group.

### Clinical Characteristics of the Participants

Participant clinical characteristics are shown in Table 1. We found no significant difference in age, gender, handedness, Edinburgh Handedness Inventory, MMSE score, or Epworth sleepiness score among the three groups. HADS, HADS-depression, and Pittsburgh Sleep Quality Inventory scores were significantly higher in the RBD-MMI group compared to the HC group. Although UPDRS-III scores were significantly higher in the RBD-MMI group relative to the HC group, there was no significant difference in scores between the RBD-MMI and RBD-N groups (*P* = 0.229). Only the scores regarding finger tapping included in UPDRS-III (item 23) in the RBD-MMI group were significantly higher than those of the RBD-N group and HC group (Table 2). This result was consistent with the above results, which were revealed by objective finger tapping assessment. Scores on the odor identification test were significantly lower in the RBD-MMI and RBD-N groups compared to the HC group. There were no significant differences between RBD-MMI and RBD-N groups in terms of disease duration or polysomnographic measures (Table 3).

**Table 1:** Clinical characteristics of RBD-MMI, RBD-N, and HC group.

**Subjects**	**RBD-MMI (A) *N* = 8**	**RBD-N (B) *N* = 15**	**HC (C) *N* = 20**	**P**	***Post-hoc* significance**
Age (years)	72.2 (4.6)	70.8 (4.0)	70.7 (3.6)	0.63	N/A
Gender	4M: 4F	8M: 7F	11M: 9F	0.97	N/A
Handedness	8R: 0L	15R: 0L	20R: 0L	N/A	N/A
Edinburgh Handedness Inventory	92.5 (11.6)	95.8 (6.6)	93.7 (19.5)	0.85	N/A
Disease duration (years)	5.6 (3.9)	4.7 (3.0)	N/A	0.54	N/A
Total UPDRS III	2.3 (2.2)	1.2 (1.6)	0.5 (0.9)	0.017	A>C^*^
MMSE	29.3 (0.9)	28.6 (1.2)	28.7 (1.7)	0.47	N/A
HADS	12.6 (8.0)	9.1 (4.8)	5.2 (5.0)	0.008	A>C^**^
HADS-anxiety	5.2 (5.9)	3.5 (2.5)	1.9 (2.3)	0.060	N/A
HADS-depression	7.3 (3.8)	5.0 (3.6)	3.3 (3.0)	0.024	A>C^*^
Odor identification test	4.2 (2.4)	4.8 (1.7)	6.8 (1.5)	0.001	A < C^**^, B < C^**^
RBDSQ-J	9.1 (1.8)	8.0 (2.5)	1.3 (1.8)	<0.001	A>C^***^, B>C^***^
Epworth sleepiness score	4.5 (3.5)	5.8 (2.9)	3.8 (2.6)	0.13	N/A
Pittsburgh sleep quality inventory	6.3 (3.8)	5.0 (2.1)	3.0 (2.5)	0.011	A>C^*^

*Data shown are mean (SD)*.

* *P < 0.05*,

** *P < 0.01*,

*** *P < 0.001. UPDRS, Unified Parkinson's Disease Rating Scale; Rt, Right; Lt, Left; MMSE, Mini-Mental State Examination; RBDSQ-J, Japanese version of the Rapid Eye Movement Sleep Behavior Disorder Screening Questionnaire; HADS, Hospital Anxiety and Depression Scale; N/A, Not Applicable*.

**Table 2:** The detailed UPDRS III scores in RBD-MMI, RBD-N, and HC group.

**Items**	**RBD-MMI (A) *N* = 8**	**RBD-N (B) *N* = 15**	**HC (C) *N* = 20**	***P***	***Post-hoc* significance**
Speech	0.12 (0.35)	0.06 (0.25)	0 (0)	0.34	N/A
Facial expression	0 (0)	0.06 (0.25)	0 (0)	0.40	N/A
Tremor at rest	0 (0)	0 (0)	0 (0)	N/A	N/A
Action or postural tremor of hands	0.12 (0.35)	0.06 (0.25)	0.05 (0.22)	0.79	N/A
Rigidity	0.25 (0.46)	0.33 (0.61)	0.2 (0.41)	0.73	N/A
Rt Finger tapping	0.62 (0.51)	0.13 (0.35)	0.05 (0.22)	<0.01	A>B^**^, A>C^***^
Lt Finger tapping	0.50 (0.53)	0 (0)	0.05 (0.22)	<0.001	A>B^***^, A>C^**^
Hand movements	0.25 (0.70)	0.13 (0.35)	0.10 (0.30)	0.69	N/A
Rapid alternative movements of hands	0 (0)	0.13 (0.35)	0 (0)	0.14	N/A
Leg agility	0.12 (0.35)	0.13 (0.35)	0 (0)	0.25	N/A
Arising from chair	0 (0)	0 (0)	0 (0)	N/A	N/A
Posture	0 (0)	0.06 (0.25)	0 (0)	0.40	N/A
Gait	0 (0)	0 (0)	0 (0)	N/A	N/A
Postural stability	0 (0)	0.06 (0.25)	0 (0)	0.40	N/A
Body bradykinesia and hypokinesia	0.12 (0.35)	0.13 (0.35)	0.05 (0.22)	0.67	N/A

*Data shown are mean (SD)*.

** *P < 0.01*,

*** *P < 0.001. UPDRS, Unified Parkinson's Disease Rating Scale; Rt, Right; Lt, Left; N/A, Not Applicable*.

**Table 3:** Polysomnographic sleep measures in the RBD-MMI and RBD-N group.

**Subjects**	**RBD-MMI**	**RBD-N**	***P***
	**(A) *N* = 8**	**(B) *N* = 15**	
Total sleep time (min)	408.9 (71.3)	405.7 (66.1)	0.91
Sleep efficiency (%)	78.9 (11.9)	77.3 (11.9)	0.76
**Latency to**			
Sleep onset (min)	12.0 (5.5)	11.1 (7.3)	0.77
REM sleep (min)	84.3 (74.8)	95.5 (45.8)	0.66
**Sleep stages (% of total sleep time)**			
N1 sleep	15.1 (6.1)	23.0 (21.5)	0.32
N2 sleep	57.9 (10.6)	57.0 (14.4)	0.87
N3 sleep	3.5 (3.5)	2.4 (3.4)	0.45
REM sleep	21.3 (8.1)	26.5 (31.5)	0.65
REM sleep without atonia (%)	47.9 (22.2)	50.1 (23.7)	0.83
Arousal index	14.4 (3.6)	14.0 (6.4)	0.85
Periodic leg movement index	11.8 (16.9)	21.4 (21.1)	0.28
Apnea-hypopnea index	4.9 (5.4)	5.5 (6.7)	0.81

*Data shown are mean (SD)*.

### ^123^I-FP-CIT Analysis

**Figure 3:**
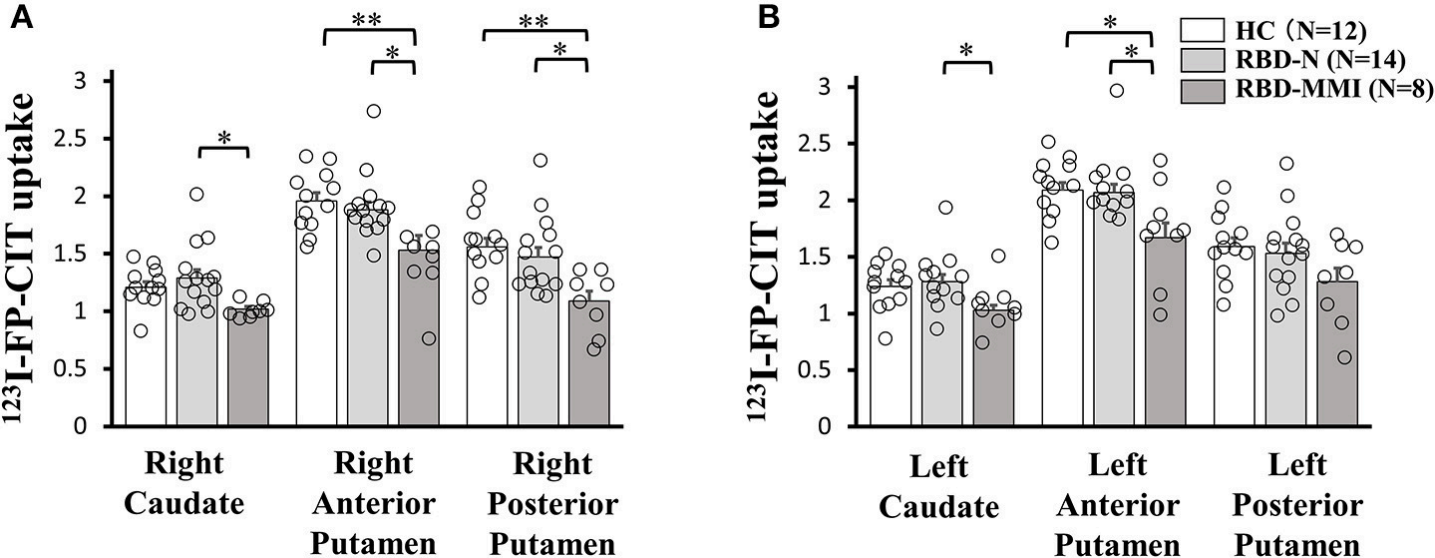
^123^I-FP-CIT uptake in the bilateral putamen and caudate of RBD-MMI, RBD-N, and HC group. ^123^I-FP-CIT uptake in the right posterior putamen and anterior putamen was significantly lower in patients of the RBD-MMI group when compared to HC subjects (**A**: right side, **B**: left side). ^123^I-FP-CIT uptake in the right posterior putamen, bilateral anterior putamen, and bilateral caudate were significantly lower in patients of the RBD-MMI group relative to those in the RBD-N group. Error bars represent the standard error of the mean. *P*-values were corrected using Tukey's multiple comparison test. ^*^P < 0.05, ^**^P < 0.01.

**Figure 4:**
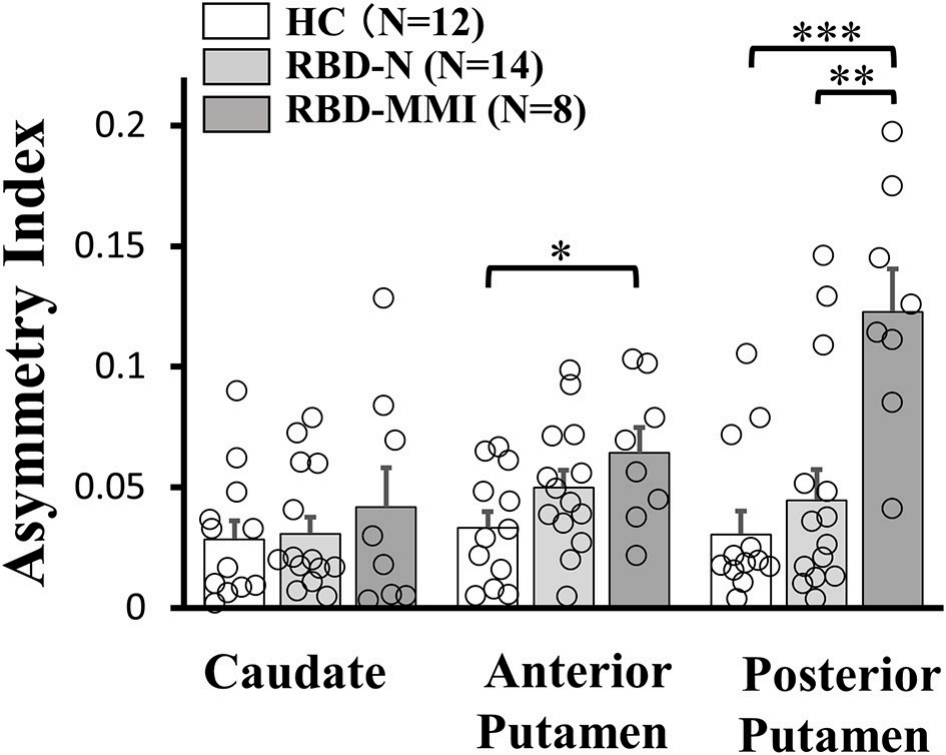
Asymmetry Index of ^123^I-FP-CIT uptake in the bilateral putamen and caudate of RBD-MMI, RBD-N, and HC group. Asymmetry Index of ^123^I-FP-CIT uptake in the posterior putamen was significantly higher in patients of the RBD-MMI group when compared to HC subjects and patients of the RBD-N group. Asymmetry Index of ^123^I-FP-CIT uptake in the anterior posterior putamen was significantly higher in patients of the RBD-MMI group relative to HC subjects. Error bars represent the standard error of the mean. *P*-values were corrected using Tukey's multiple comparison test. ^*^P < 0.05, ^**^P < 0.01, ^***^P < 0.001.

A one-way ANOVA followed by a *post-hoc* test revealed that ^123^I-FP-CIT uptake in the right posterior putamen and bilateral anterior putamen were significantly reduced in the RBD-MMI group compared to the HC group (Figure 3). Moreover, ^123^I-FP-CIT uptake in the right posterior putamen, bilateral anterior putamen, and bilateral caudate were significantly reduced in the RBD-MMI group compared to the RBD-N group. Mean ^123^I-FP-CIT uptake in the right and left posterior putamen of the RBD-MMI group was 30.2 and 19.5% lower than the mean uptake of these regions in the HC group, respectively. Similarly, mean uptake in the right and left anterior putamen of the RBD-MMI group was 22.0 and 20.1% lower than in the HC group, respectively. Mean uptake in the right and left caudate of the RBD-MMI group was also 15.8 and 17.0% lower than in the HC group, respectively. On the other hand, mean uptake in the right and left posterior putamen of the RBD-N group was only 4.1 and 3.8% lower than that of the HC group, respectively. The RBD-N group also displayed uptake that was only 4.6 and 1.0% lower in the right and left anterior putamen relative to the HC group, respectively. A significant difference in the asymmetry index of the posterior putamen was observed between the RBD-MMI and HC group (Figure 4).

### RsfMRI Analysis

Actual measures of functional connectivity between seed regions in each three group were summarized (Supplementary Tables 1–3). The RBD-MMI group showed decreased functional connectivity between the striatum and cerebral cortex when compared to the HC group (Table 4). The RBD-MMI group also showed increased functional connectivity between cerebellum and cerebral cortex compared to the RBD-N group. On the other hand, the RBD-N group showed decreased functional connectivity between the striatum and cerebral cortex when compared to the HC group. The mean absolute head motion during functional MRI acquisition did not significantly differ among the three groups (0.23 ± 0.14, 0.22 ± 0.11, and 0.16 ± 0.076 mm in the HC, RBD-N, and RBD-MMI groups, respectively, *P* = 0.422 in one-way ANOVA).

**Table 4:** Group differences in resting state functional connectivity.

	**Connection**		***T*-value**	**P-FDR**
	**ROI 1**	**ROI 2**		
RBD-MMI > HC	Rt Anterior Putamen	Rt SPL	−4.31	0.0070
	Lt Anterior Putamen	Rt SPL	−3.44	0.0205
	Lt Caudate	Rt SPL	−3.78	0.0132
RBD-MMI > RBD-N	Rt Cerebellum lobule VIII	Rt S1	3.42	0.0415
	Lt Cerebellum lobule VIII	Rt S1	3.99	0.0226
	Lt Cerebellum lobule VIII	Lt S1	3.38	0.0301
	Lt Cerebellum lobule VIII	Rt PM	3.53	0.0301
RBD-N > HC	Rt Anterior Putamen	Rt SPL	−3.86	0.0078
	Lt Anterior Putamen	Rt SPL	−4.71	0.0014

*Rt, Right; Lt, Left; SPL, Superior parietal lobule; S1, Primary somatosensory cortex; PM, Premotor cortex*.

### Correlation Analysis

A part of finger tapping parameters was significantly correlated with ^123^I-FP-CIT uptake in the striatum (Tables 5A,B). On the other hand, a part of finger tapping parameters score and finger tapping scores included in UPDRS-III were significantly correlated with Fisher-transformed *Z*-values of functional connectivity between some seed regions (Table 6). There was no significant correlation between UPDRS-III total score and ^123^I-FP-CIT uptake in the striatum or Fisher-transformed *Z*-values of functional connectivity.

**Table 5:** Correlation coefficient between ^123^I-FP-CIT uptake in the striatum and finger tapping or UPDRS-III score in all subjects.

**(A)**	**Lt Post. Putamen**	**Lt Ant. Putamen**	**Lt Caudate**
Rt Amplitude	0.16	0.43^*^	0.38^*^
Rt Peak open speed	0.27	0.47^**^	0.33
Rt Peak close speed	0.075	0.37^*^	0.31
**UPDRS-III score**			
Total score	0.14	0.025	−0.33
Rt finger tapping	0.015	−0.14	−0.30
**(B)**	**Rt Post. putamen**	**Rt Ant. putamen**	**Rt Caudate**
Lt Amplitude	0.34^*^	0.34^*^	0.25
Lt Peak open speed	0.26	0.29	0.24
Lt Peak close speed	0.30	0.35^*^	0.27
**UPDRS-III score**			
Total score	0.009	−0.049	−0.16
Lt finger tapping	−0.21	−0.11	−0.27

*Measures represent Pearson's correlation coefficient. Rt, Right; Lt, Left; Post, Posterior; Ant, Anterior; UPDRS, Unified Parkinson's Disease Rating Scale*.

* *P < 0.05*,

** *P < 0.01*.

**Table 6:** Correlation coefficients for finger tapping and altered functional connectivity in all subjects.

	**Connection**		***r***	***P***
	**ROI 1**	**ROI 2**		
**Finger tapping parameters**				
Rt Amplitude	Lt Caudate	Rt SPL	0.36	0.0208
	Rt Anterior Putamen	Rt SPL	0.30	0.0489
Lt Amplitude	Lt Caudate	Rt SPL	0.43	0.0079
	Rt Anterior Putamen	Rt SPL	0.39	0.0238
	Lt Anterior Putamen	Rt SPL	0.32	0.0350
Rt Peak open speed	Rt Anterior Putamen	Rt SPL	0.32	0.0426
	Lt Anterior Putamen	Rt SPL	0.32	0.0368
Lt Peak open speed	Rt Anterior Putamen	Rt SPL	0.40	0.0092
	Lt Anterior Putamen	Rt SPL	0.31	0.0444
Rt Peak close speed	Rt Anterior Putamen	Rt SPL	0.43	0.0045
Lt Peak close speed	Rt Anterior Putamen	Rt SPL	0.50	0.008
	Lt Anterior Putamen	Rt SPL	0.46	0.0024
**UPDRS-III score**				
Rt finger tapping	Lt Cerebellum lobule VIII	Lt S1	0.32	0.0350
Lt finger tapping	Lt Caudate	Rt SPL	−0.40	0.0092
	Lt Cerebellum lobule VIII	Rt S1	0.30	0.0489

*Rt, Right; Lt, Left; SPL, Superior parietal lobule; S1, Primary somatosensory cortex; PM, Premotor Cortex; UPDRS, Unified Parkinson's Disease Rating Scale*.

## Discussion

In the present study, we successfully employed finger tapping parameters including amplitude, peak open speed, and peak close speed to identify patients with RBD with mild motor impairment and showed that such patients presented nigrostriatal dopaminergic dysfunction, as well as alterations in resting state cortico-striatal and cortico-cerebellar networks.

We revealed that eight (34%) out of 23 patients with RBD presented mild motor impairment (RBD-MMI group), as defined by six finger tapping parameters. In two patients, five parameters were <2 SD of the mean value of those of the HC group. In one patient, three parameters were <2 SD of the mean value of those of the HC group. In four patients, two parameters were <2 SD of the mean value of those of the HC group. In one patient, one parameter was <2 SD of the mean value of that of the HC group. Moreover, seven patients exhibited lower right amplitude, six patients exhibited lower left close speed, and five patients exhibited lower left amplitude. Right amplitude may be a global measure of mild motor impairment. Our study revealed heterogeneous motor impairment patterns in patients with RBD. In the previous study, motor function in patients with RBD was assessed with UPDRS part-III, Perdue Pegboard, Timed Up and Go and Alternate tap test (5, 6, 37). However, there was no study to investigate finger taping parameters in patients with RBD. The reason we selected finger tapping to evaluate mild motor impairment in RBD is that finger tapping amplitude and speed are important factors to evaluate Parkinsonism as they are included in UPDRS-III scoring system. We speculate that finger tapping amplitude and speed may not necessarily decrease in parallel at the early stage of Parkinson's disease. This idea is based on the previous study in which the pathophysiology of lower amplitude and lower speed in finger tapping in Parkinson's disease may be different (8). In that study, finger tapping amplitude was more impaired than speed and finger tapping speed was more improved than amplitude with dopaminergic agent. However, it is unclear that which of amplitude or speed is more impaired in the very early stage of Parkinson's disease. We think that, to detect mild motor impairment which may reflect the prodromal stage of Parkinson's disease, separate assessment for finger tapping amplitude and speed are necessary. Moreover, although we also separately evaluated finger tapping speed as open and close speed, this is based on the difference of muscle activity between during opening and closing phase in finger tapping. During fast repetitive movement without physical stop, antagonist muscle activity to stop ongoing movement (in the present study, near at the end phase of finger opening) works as agonist muscle for following movement (finger closing) (38). This flexion slowness reflects a characteristic of Parkinson's disease which is known as implementing dysfunction of agonist muscle activity (39, 40). The findings that finger tapping amplitude and close speed was more impaired than open speed may suggest that our criteria for mild motor impairment detects very early motor manifestation in the prodromal stage of Parkinson's disease. No patient exhibited progressive decrement of amplitude and speed, important features that define bradykinesia (41). Our results may also indicate that progressive decrement of finger tapping parameter is followed by the reduction of finger tapping parameter in the prodromal stage of Parkinson's disease.

While RBD-MMI and RBD-N group exhibited no significant differences in UPDRS-III total score, all finger tapping parameters were significantly reduced in the RBD-MMI group when compared to the RBD-N group. Although the UPDRS-III finger tapping scores were also significantly higher in the RBD-MMI group, mean scores were very low. The objective finger tapping evaluation may be more sensitive in detecting mild motor impairment than UPDRS-III score. Another strength of our study is that evaluation is not dependent on the clinical experience of movement disorder. Moreover, patients in the RBD-MMI group presented mild depression, lower sleep quality, and olfactory dysfunction. Depression, olfactory dysfunction, and sleep disturbance are non-motor symptoms in Parkinson's disease (42–44). They may have neurodegeneration which is similar to that in Parkinson's disease. There was no difference in polysomnographic measures between the RBD-MMI group and RBD-N group. Finger tapping impairment was assumed to be independent from the pathophysiology of RBD itself.

The RBD-MMI group revealed lower ^123^I-FP-CIT uptake in the right posterior putamen, bilateral anterior putamen, and bilateral caudate when compared to the HC group or RBD-N groups. There was no significant difference in^123^I-FP-CIT uptake in the left putamen among three groups. Low number of our subjects may affect the results. The reduction in ^123^I-FP-CIT uptake was graded (posterior putamen > anterior putamen > caudate) in patients of the RBD-MMI group. This pattern of striatal dopaminergic function, affecting ventrolateral projections to the dorsal putamen rather than dorsomedial projections to the caudate, is comparable to that seen in Parkinson's disease (45, 46). In the early stage of Parkinson's disease (UPDRS-III score 14.8 ± 4.9), the reduction in ^123^I-FP-CIT uptake (relative to healthy controls) is 52% in the putamen (47). In another study of early stage Parkinson's disease (UPDRS-III score 14 ± 7.3), the reduction in ^123^I-β-CIT uptake (relative to healthy controls) is 58% in the contralateral putamen and is 44% in the ipsilateral putamen (48). On the other hand, a previous study revealed that in patients with RBD (UPDRS-III score 3.13 ± 3.63), the mean ^123^I-FP-CIT binding was decreased by 8.0% in the putamen (16). In another study, ^123^I-ioflupane uptake in the putamen was 13.2% lower in RBD group (UPDRS-III score 3.3 ± 3.5) than that in the normal controls and 30.8% higher than that in patients with Parkinson's disease (23). In the current study, the RBD-MMI group exhibited ^123^I-FP-CIT uptake in the putamen that was 19.5–30.5% lower than that observed in the HC group. Nigrostriatal dopaminergic dysfunction in patients with RBD-MMI is less severe than disruptions observed in the early stages of Parkinson's disease, but is a little more severe than that in previously reported patients with RBD.

Asymmetry of ^123^I-FP-CIT uptake in the posterior and anterior putamen was higher in the RBD-MMI group. Higher asymmetry index of ^123^I-FP-CIT uptake in the posterior putamen was also a characteristic finding in Parkinson's disease (47). Decreased dopamine transporter uptake and higher asymmetry index in the striatum of patients with RBD is associated with high risk of developing a neurodegenerative disease (16, 17, 49, 50). Patients with RBD-MMI may have a higher conversion risk to Parkinson's disease or dementia with Lewy bodies.

The RBD-MMI group exhibited decreased cortico-striatal functional connectivity compared to the HC group and increased cortico-cerebellar functional connectivity compared to patients with the RBD-N group. The RBD-N group also exhibited decreased cortico-striatal functional connectivity compared to the HC group. In the previous study, cortico-striatal functional connectivity was increased in off-state patients with early Parkinson's disease and decreased in on-state (19, 51). Although longitudinal follow up is necessary, when patients with RBD develop Parkinson's disease, cortico-striatal functional connectivity may turn to be increased. A previous meta-analysis of functional MRI studies on healthy subjects demonstrated functional connectivity between the anterior putamen or caudate and the posterior parietal cortex (52). The posterior parietal cortex (including the SPL) is involved in sensorimotor integration (53). In the present study, patients in the RBD-MMI and RBD-N group showed decreased functional connectivity between the anterior putamen or caudate and SPL. This suggests that sensorimotor integration may already be altered in patients with RBD, which may reflect early neurodegeneration. There was no significant difference in ^123^I-FP-CIT uptake in the anterior putamen and caudate between the RBD-N group and HC group. The cortico-striatal functional connectivity alteration may be dopamine-independent alteration. The combination of mild nigrostriatal dopaminergic dysfunction and decreased cortico-striatal connectivity may be relevant for mild motor impairment in patients with RBD.

The present study also showed that, relative to patients in the RBD-N group, patients in the RBD-MMI group exhibited increased functional connectivity between cerebellar lobule VIII and S1 or PM. Increased cerebellar-cortical connectivity was reported in patients with early Parkinson's disease (54). In functional MRI study in Parkinson's disease, the activity of cerebellum was increased during automated movements, which may reflect compensatory function of cerebellum (55). Increased cerebellar-cortical functional connectivity in the RBD-MMI group may represent compensation for mild nigrostriatal dopaminergic dysfunction. However, the functional connectivity between cerebellar lobule VIII and S1was positively correlated with the UPDRS-III finger tapping score. Increased cerebellar-cortical connectivity may also represent pathological connectivity. There were two previous studies which investigated resting state functional connectivity in patients with RBD. One study revealed functional connectivity alteration between the left substantia nigra and left putamen, and the right substantia nigra and right cuneus/precuneus, and the right substantia nigra and right superior occipital gyrus in patients with RBD compared to healthy control subjects (56). Another study revealed basal ganglia network alteration in patients with RBD, which had no significant difference compared to patients with Parkinson's disease (23). The results in the present study were different from those in the two previous studies. Several reasons which explain the different results include the difference of the methodology, the number of subjects, and the heterogeneity of patients with RBD. We used ROI to ROI analysis with seed regions which were related to sensorimotor function, the two previous studies used seed to voxel analysis or independent component analysis. The lower number of subjects in our study than the two studies may relate to the results. Moreover, the heterogeneity of patients with RBD may contribute to the results. Some patients with long-standing RBD doesn't develop neurodegenerative disorders for 10–12 years, other patients developed neurodegenerative disorders within 4 years after the diagnosis of RBD (57, 58). Functional connectivity alteration may also be heterogeneous in patients with RBD.

We investigated the associations between finger tapping parameters and ^123^I-FP-CIT uptake or functional connectivity. A part of finger tapping parameters were significantly correlated with contralateral ^123^I-FP-CIT uptake or resting state functional connectivity. On the other hand, UPDRS-III finger tapping scores were significantly correlated with only resting state functional connectivity. Our results show that finger tapping was affected by not only nigrostriatal dopaminergic dysfunction but resting state functional connectivity. There is no study which revealed a significant correlation between motor function and ^123^I-FP-CIT uptake or resting state functional connectivity in patients with RBD. The quantitative evaluation of motor function may be a sensitive method to detect a significant association between finger tapping and neuroimaging measures. In fact, the evaluation of finger tapping with UPDRS-III depends on the subjective assessment of examiner. Interestingly, brain measures of not posterior putamen but anterior putamen or caudate were correlated with motor function. Posterior putamen is sensorimotor related area and caudate and anterior putamen are associative striatum (59). ^18^F-FP-CIT uptake in anterior putamen was related to attention, working memory, executive function, and visuospatial functions in patients with Parkinson's disease (60). Since we didn't perform enough neuropsychological tests, it is unclear whether non-motor function affects finger tapping impairment.

There are several limitations to our study. We originally defined the criteria for mild motor impairment. Strictly, we should investigate whether our criteria really reflect mild motor impairment. If patients with mild motor impairment highly develop Parkinson's disease or dementia with Lewy bodies and each finger tapping parameters become worse at that time, mild motor impairment we defined will be a useful marker to evaluate motor function in the prodromal stage of Parkinson's disease or dementia with Lewy bodies. Since we focused on hand motor dysfunction, we may overlook other kinds of mild motor impairment such as gait disturbance. In fact, the UPDRS-III rigidity score was higher in the RBD-N group than in the RBD-MMI group. The sample size was as small as 20–23 cases in each group, therefore, further larger and longitudinal studies are required to confirm our results.

In conclusion, some patients with RBD present mild motor impairment and such have mild nigrostriatal dopaminergic dysfunction and cortico-striatal and cortico-cerebellar functional connectivity alteration. They may have higher risk of conversion to neurodegenerative diseases.

## Ethics Statement

This study was carried out in accordance with the recommendations of the Ethics Committee of the Nagoya City University with written informed consent from all subjects. All subjects gave written informed consent in accordance with the Declaration of Helsinki. The protocol was approved by the Ethics Committee of the Nagoya City University.

## Author Contributions

YU, NO, TO, and MN contributed conception and design of the study. AF, HK, NA, and KS contributed execution of the experiments. YS and AK performed the statistical analysis. GY wrote the first draft of the manuscript. All authors contributed to manuscript revision, read, and approved the submitted version.

### Conflict of Interest Statement

^123^I-FP-CIT was provided as DaTSCAN by Mediphysics (Tokyo, Japan). AK and YS were employed by Hitachi Ltd. The remaining authors declare that the research was conducted in the absence of any commercial or financial relationships that could be construed as a potential conflict of interest.

## Abbreviations

^123^I-FP-CIT: ^123^I-2β-carbomethoxy-3β-(4-iodophenyl) nortropane
AAL: Automated Anatomical Labeling
HC: healthy control
IPL: inferior parietal lobule
M1: primary motor cortex
MMI: Mild motor impairment
MMSE: Mini-Mental State Examination
MNI: Montreal Neurological Institute
MPRAGE: magnetization prepared-rapid acquisition gradient echo
PM: premotor cortex
RBD: REM sleep behavior disorder
RBD-MMI: RBD with mild motor impairment
RBD-N, RBD without mild motor impairment: or normal motor function
RBDSQ-J: Japanese version of the RBD-Screening Questionnaire
ROI: region of interest
S1: primary sensory cortex
SMA: supplementary motor area
SPL: superior parietal lobule
TE: echo time
UPDRS-III: Unified Parkinson's Disease Rating Scale part III
VOIs: volumes of interests.
