# Supplementary material for: Nigrostriatal Dopaminergic Dysfunction and Altered Functional Connectivity in REM Sleep Behavior Disorder With Mild Motor Impairment
Source: Front Neurol. 2019 Jul 26;10:802. doi: 10.3389/fneur.2019.00802 (PMC6677031; doi:10.3389/fneur.2019.00802)
Supplement: Supplementary file 1 [file Table_1.DOCX]

Supplementary Table 1 Fisher’ transformed Z vakues between each seed regions in the HC subjects

HC, Healthy Control; Thal, Thalamus; SPL, Superior Parietal Lobe; SMA, Supplementary Motor Area; S1, Primary Sensory Cortex; Post Put, Posterior Putamen; Ant Put, Anterior Putamen; PM, Premotor Cortex; M1, Primary Motor Cortex; C, Cerebellum; Caud, Caudate; IPL, Inferior Parietal Lobe;
